# Roots of symptom-free leguminous cover crop and living mulch species harbor diverse *Fusarium* communities that show highly variable aggressiveness on pea (*Pisum sativum*)

**DOI:** 10.1371/journal.pone.0191969

**Published:** 2018-02-14

**Authors:** Adnan Šišić, Jelena Baćanović-Šišić, Petr Karlovsky, Raphaël Wittwer, Florian Walder, Enio Campiglia, Emanuele Radicetti, Hanna Friberg, Jörg Peter Baresel, Maria R. Finckh

**Affiliations:** 1 Department of Ecological Plant Protection, University of Kassel, Witzenhausen, Germany; 2 Section of Organic Plant Breeding and Agrobiodiversity, University of Kassel, Witzenhausen, Germany; 3 Department of Molecular Phytopathology and Mycotoxin Research, University of Göttingen, Göttingen, Germany; 4 Plant-Soil-Interactions Group, Institute for Sustainability Science, Agroscope, Zurich, Switzerland; 5 Department of Agricultural and Forestry Sciences (DAFNE), University of Tuscia, Viterbo, Italy; 6 Department of Forest Mycology and Plant Pathology, Swedish University of Agricultural Sciences, Uppsala, Sweden; 7 Institute for Plant Nutrition, Center of Life and Food Sciences Weihenstephan, Technical University Munich, Freising-Weihenstephan, Germany; Tallinn University of Technology, ESTONIA

## Abstract

Leguminous cover crop and living mulch species show not only great potential for providing multiple beneficial services to agro-ecosystems, but may also present pathological risks for other crops in rotations through shared pathogens, especially those of the genus *Fusarium*. Disease severity on roots of subterranean clover, white clover, winter and summer vetch grown as cover crop and living mulch species across five European sites as well as the frequency, distribution and aggressiveness to pea of *Fusarium* spp. recovered from the roots were assessed in 2013 and 2014. Disease symptoms were very low at all sites. Nevertheless, out of 1480 asymptomatic roots, 670 isolates of 14 *Fusarium* spp. were recovered. The most frequently isolated species in both years from all hosts were *F*. *oxysporum* and *F*. *avenaceum* accounting for 69% of total isolation percentage. They were common at the Swiss, Italian and German sites, whereas at the Swedish site *F*. *oxysporum* dominated and *F*. *avenaceum* occurred only rarely. The agressiveness and effect on pea biomass were tested in greenhouse assays for 72 isolates of six *Fusarium* species. Isolates of *F*. *avenaceum* caused severe root rot symptoms with mean severity index (DI) of 82 and 74% mean biomass reduction compared to the non-inoculated control. *Fusarium oxysporum* and *F*. *solani* isolates were higly variable in agressiveness and their impact on pea biomass. DI varied between 15 and 50 and biomass changes relative to the non-inoculated control -40% to +10%. Isolates of *F*. *tricinctum*, *F*. *acuminatum* and *F*. *equiseti* were non to weakly agressive often enhancing pea biomass. This study shows that some of the major pea pathogens are characterized by high ecological plasticity and have the ability to endophytically colonize the hosts studied that thus may serve as inoculum reservoir for susceptible main legume grain crops such as pea.

## Introduction

In the past 10 to 15 years, there has been a tendency in Europe to switch from cereal based cropping systems, with winter and summer fallow, to cropping systems which combine greater crop diversity with maximum soil cover [1,2]. As a consequence, an increasing number of different legumes, especially clovers and vetches, are being grown in rotations or associations with cereals [3–5]. Legumes provide multiple ecological services to agricultural systems. The main benefit of their inclusion in cropping systems is their ability to fix atmospheric nitrogen [6,7]. When planned properly in terms of adequate diversity and appropriate density, legume based species mixtures and crop rotations may also contribute to mobilization and remineralization of nutrients [8], improved soil structure [9], increased water infiltration and prevention of soil erosion [10,11] as well as weed suppression [12]. In addition to the increased use of legumes as cover crops (CCs) and living mulches (LMs) there is a growing demand for grain legume cash crops that are an important protein source for animal and human nutrition. Under European conditions these are mainly pea (*Pisum sativum*) and faba bean (*Vicia faba*) that are comonly grown in rotation with cereals [13,14]. Despite their agronomic value, legume production in Europe, in particular pea, has been declining mainly due to problems with soil-borne pathogens and weeds [15–17].

Many of the leguminous species that can be used as CCs and LMs share important soil-borne-pathogens among each other and with some important main crops such as pea [18]. Thus, changes towards legume rich crop rotations may increase the prevalence of such pathogens within the agro-ecosystems leading to increased disease pressure. As the success of legume production and its ability to provide ecosystem services depends crucially on legume root health, keeping the root system productive in such, legume intensive growing systems, becomes especially challenging. Therefore, the potential agronomic and environmental benefits of leguminous CCs and LMs can only become effective if the pathological risks are thoroughly assessed and solutions for potential problems identified.

Species of the genus *Fusarium* are of particular importance as they can efficiently spread along crop rotations, and often infect a wide range of plants under diverse environmental conditions. Mainly *F*. *oxysporum*, *F*. *solani*, *F*. *avenaceum*, *F*. *redolens* and *F*. *culmorum* are associated with roots of leguminous crops [18–21], and usually occur as a pathogen complex rather than alone. Prevalence, dominance and importance of the single pathogens may vary greatly depending on location, climate, and agricultural practice [22] and some shifts in importance have occurred over time. For example, *F*. *solani* has been described as one of the major and most aggressive pathogens of pea (*Pisum sativum* L.) during the 1980s and 1990s [23,24]. In a more recent survey conducted in Germany, *F*. *redolens* together with *F*. *oxysporum* and *F*. *avenaceum* dominated among *Fusarium* spp. [19]. Similarly, in disease surveys conducted in North Dakota, USA and central Alberta, Canada, Chittem et al. [25] and Holtz et al. [26] reported that *F*. *avenaceum* dominated the complex of *Fusarium* species associated with root rot of pea and lupines (*Lupinus angustifolius*). In both studies addressing pea root rot [19,25], members of the *F*. *oxysporum* species complex were isolated two to three times more often than *F*. *solani* from diseased roots.

While pathogens involved in the root rot disease complex of main legume crops such as pea have been extensively studied [25,27], there is a lack of information about the composition of species involved, geographical distribution and abundance of *Fusarium* spp. colonizing perspective cover crop and living mulch species. In order to understand the importance of root infecting *Fusarium* spp., root samples of subterranean clover, white clover, winter vetch, and summer vetch grown in rotation or association with wheat were collected in 2013 and 2014 from five field sites across Europe. The main objectives of the study were to assess root rot symptom severity and to characterize diversity, frequency and possible underlying patterns of host preference and geographical distribution of *Fusarium* spp. associated with roots of these four perspective CC/LM crops. A further objective was to evaluate the aggressiveness of the predominantly found *Fusarium* species on pea, in order to determine the potential role of clover and vetch species as alternate hosts of pathogens of importance to the a main legume crop. In this article, the term aggressiveness refers to relative ability of pathogen/isolate to colonize and cause damage to plants [28].

## Material and methods

### Sampling sites and plant material

Roots of subterranean clover (*Trifolium subterraneum* L.), white clover (*T*. *repens* L.), winter vetch (*Vicia villosa* Roth) and summer vetch (*V*. *sativa* L.) intercropped with wheat as living mulch or grown as cover crops after wheat were collected during 2013 and spring 2014. The samples were collected from field experiments set up jointly in five European sites representing different agro-climatic zones and soil conditions: University of Tuscia (Localita' Riello, Viterbo, Italy), Agroscope (Reckenholz, Zürich, Switzerland), Technical University Munich (Freising, Munich, Germany), University of Kassel (Witzenhausen, Germany), and Swedish University of Agricultural Sciences (Upsala, Sweden) (Table 1). Plant samples were collected in a W pattern across the field plots at each site, washed under running tap water and individual plants were evaluated for root rot disease severity using a 0–5 rating scale modified from Aldoud et. al. [29] and Flett et. al. [30] as follows: 0 = healthy, 1 = lateral root discoloration (tap root remains healthy), 2 = less than 1/3 of tap root discolored, 3 = 1/3 to 2/3 of tap root discolored, 3.5 = tap root rotted away but new lateral roots are growing, 4 = more than 2/3 of tap root discolored and no new lateral roots are growing, 4.5 = tap root rotted away, 5 = plant dead. The roots collected at the Italian site and white clover samples from southern Germany (Munich) were not subjected to disease severity assessments due to the overall healthy appearance of the plants. Timing of plant sample collection varied across the sites and was adjusted to site specific climatic and cultural practices such as crop rotation, tillage and planting dates (Table 1).

**Table 1:** Site-specific pedo-climatic characteristics, plant species, sampling dates and number of root samples processed per site and year.

Site^1^, climate zone^2^ [31]	Temp.^3^ (°C)	Ppt.^4^ (mm)	Soil Type	pH	% OM	Plant species^5^, use^6^ (2013/14)	2013			2014		
							N^7^	n^8^	Sampling time	N	n	Sampling time
**Italy** 42°25'N, 12°05'E (MDN)	11.6	845	Typic Xero-fluvent	6.7	1.2	Subclover LM/CC	-	120	26. Apr	-	120	23. Apr
						W. vetch CC	-	-	-	-	120	23. Apr
**Switzerland** 47°30'N, 8°55'E (CON)	9.5	1111	Hapludalf	7.1	2.0	Subclover LM/CC	657	120	01. July	1101	120	14. Apr
						W. vetch CC	-	-	-	476	120	14. Apr
**South Germany** 11° 41'E, 48° 23' N (CON)	8.3	805	Cambisol	6.9	1.6	Subclover LM	152	80	17. Dec	-	-	-
						W. clover LM	-	80	17. Dec	-	-	-
						W. vetch CC	98	80	17. Dec	-	-	-
**Central Germany** 51°22'N, 9°54'E (ATN)	9.4	644	Typic Haplu-dalf	6.2	2.0	Subclover LM/LM	292	80	30. Oct	315	80	16. June
						W. clover LM/LM	305	80	30. Oct	293	80	16. June
						S. vetch CC	80	80	30. Oct	-	-	-
**Sweden** 59°49’N 17°42’E (NEM)	8.2	598	Inceptisol	5.7	5.3	W. clover LM/CC	160	40	23. Oct	320	40	14. Apr
						W. vetch CC	160	-	-	-	40	14. Apr

^1^Experimental fields of: Italy = University of Tuscia, Localita' Riello, Viterbo; Switzerland = Agroscope, Reckenholz, Zurich; Southern Germany = Technical University Munich, Freising, Munich; Central Germany = University of Kassel, Witzenhausen; Sweden = Swedish University of Agricultural Sciences, Upsala

^2^MDN = Mediterranean North, CON = Continental, ATN = Atlantic North, NEM = Nemoral

^3^Average annual temperature

^4^Average annual precipitation

^5^Subclover = subterranean clover, W. vetch = winter vetch, W. clover = white clover, S. vetch = summer vetch

^6^LM = living mulch species, CC = cover crop species

^7^Number of roots sampled at each site and assessed for disease severity

^8^Number of roots used for isolation and identification of root infecting fungi.

After the assessments, samples were transferred to University of Kassel and stored at -20°C until further use. In total, 2517 roots of subterranean clover, 1078 of white clover, 734 of winter vetch and 319 roots of summer vetch were collected and assessed for disease severity over the two sampling years. Summer vetch was grown exclusively in 2013 at KU. Roots of subterranean clover, white clover and winter vetch from southern Germany were only available in 2013 (Table 1, S1 Table).

### Identification of fungi

In both years, sub-samples of collected plants, representing the entire field at each site, were selected for the isolation and identification of root associated fungi (Table 1). Roots were again thoroughly washed under running tap water, surface sterilized with 3% sodium hypochlorite for 10 s, rinsed in distilled water and placed on filter paper under a laminar flow hood for 1 h to dry. Three approximately 1 cm long pieces were selected randomly, placed in Petri dishes containing Coon´s medium (4 g/l Maltose, 2 g/l KNO_3_, 1.20 g/l MgSO_4_ x 7 H_2_O, 2.68 g/l KH_2_PO_4_, and 20 g/l agar) and incubated under alternating cycles of 12 h blacklight blue (BLB) fluorescent light (F40; range 315–400 nm with the peak at 365 nm) and 12 h darkness. Fungal colonies developing from the root pieces were sub-cultured separately in Petri dishes containing half-strength potato dextrose agar (19 g/l Difco PDA and 10 g/l agar).

Pure cultures were obtained either through single spore transfers or hyphal tipping, and each isolates was examined microscopically. *Fusarium* colonies were than transferred to PDA and Synthetic Nutrient-Poor Agar (SNA, [32] and identified to the species level based on cultural appearance (colony color and pigmentation) and microscopic examination of conidiogenous cells using the morphological key of Leslie and Summerell [33].

Morphological identities of randomly selected isolates of *F*. *avenaceum* (n = 5), *F*. *oxysporum* (n = 5), *F*. *culmorum* (n = 5), *F*. *graminearum* (n = 2), *F*. *tricinctum* (n = 5), *F*. *acuminatum* (n = 5) and *F*. *equiseti* (n = 5), was confirmed by real-time PCR. DNA was extracted from freeze-dried mycelia using a CTAB method of Brandfass and Karlovsky [34]. Real-time PCR was carried out under conditions optimized for each species according to the methods described in Dastjerdi [35] using following primer sets: FaF and FaR [36] for *F*. *avenaceum*, CLOX1 and CLOX2 [37] for *F*. *oxysporum*, OPT18 F and OPT18 R [38] for *F*. *culmorum*, Fg16NF and Fg16NR [39] for *F*. *graminearum*, FP82F and FP82R [40] for *F*. *poae*, Tri1 F and Ttri2 R [41] for *F*. *tricinctum*, and 198F2 and 198R1 [42] for *F*. *equiseti*. Preliminary studies showed that the primer pair Tri1 F and Tri2 R described by Kulik [41] as specific for *F*. *tricinctum* also generated products with the genomic DNA of *F*. *acuminatum*, and thus could not distinguish between the two species. Therefore, to confirm identity of the species, in addition to the expected positive signal in the qPCR assay using the aforementioned primer pair, separation was based on size and shape of micro- and macroconidia, morphological characteristics of conidiogenous cells, colony characteristics and growth rate as described above.

The identity of the selected *F*. *solani* isolates, including additional *F*. *avenaceum* isolates, was confirmed by the results of the translation elongation factor 1-alpha (*tef1*) gene sequences. For this purpose the *tef1* gene was amplified using primer pairs EF1 and EF2 described by O’Donnell et al. [43]. The PCR reactions were performed with a peQ STAR Thermocycler (96 Universal Gradient). The PCR mixture contained 5 μl of reaction buffer (16 mM (NH_4_)_2_SO_4_; 67 mM Tris-HCl; 0.01% (v/v) Tween-20, pH: 8.8 at 25°C), 2 μl of 2 mM of MgCl_2_, 1.5 μl of 0.15 mM dNTP’s (Bioline, Lukenwalde, Germany), 0.75 μl of each primer (0.3 μM), 0.05 μl of 0.25 Unit BIOTaq DNA polymerase (Bioline, Luckenwalde, Germany), 3 or 6 μl of diluted genomic DNA, and double distillated water to make total reaction volume of 25 μl.

Conditions for amplification were an initial denaturation step of 3 min at 95°C, followed by 30 cycles of 1 min at 94°C (denaturalization), 45 s at 59.1°C (annealing), 1 min at 72°C (extension) and a final extension cycle at 72°C for 5 min. The amplified DNA fragments were purified using 70% isopropanol precipitation, rinsed with 70% (v/v) ethanol, air dried, re-suspended in double distillated water, and sequenced (LGC Genomics, Berlin, Germany) in both directions using the PCR primers. The chromatogram of the *tef1* sequence for each *Fusarium* species was inspected visually and sequence reads edited when necessary. The sequences were then used as queries for the Fusarium-ID v. 1.0 database [44], and the *Fusarium* MLST database (http://www.cbs.knaw.nl/fusarium) [45]. Positive identification rate for the isolates of *F*. *solani* and *F*. *avenaceum* was >99%. These sequences were submitted to the GenBank database under following accession numbers: KY128330—KY128334; KY556472—KY556474; KY556476—KY556479; KY556484—KY556487 for *F*. *solani*, and MG674543—MG674569; MG674571—MG674573 for *F*. *avenaceum*.

### Aggressiveness of selected *Fusarium* isolates

To evaluate aggressiveness of the most common species recovered from the sampled roots, 65 isolates of six *Fusarium* species were used in a greenhouse assay. This study included 15 isolates of *F*. *oxysporum*, 15 isolates of *F*. *avenaceum*, 24 isolates of *F*. *solani*, 8 isolates of *F*. *acuminatum*, 2 isolates of *F*. *tricinctum* and 1 isolate of *F*. *equiseti*. In addition, one isolate of *F*. *oxysporum* recovered from pea roots, and one *F*. *avenaceum* and three *F*. *equiseti* isolates from wheat roots were included in the experiment. Besides the non-inoculated (negative) control treatment, two isolates of *F*. *solani* f. sp. *pisi* (GenBank accession numbers KY556491 and KY556459) collected from pea roots and known to be pathogenic on pea served as positive control. Spring field pea variety Santana served as a model plant (S3 Table).

*Fusarium solani* isolates for inoculum production were grown on half-strength PDA agar and the isolates of the remaining *Fusarium* spp. were grown on SNA agar. The plates with fungal inoculum were incubated 10–15 days at 20°C under alternating cycles of 12 h BLB fluorescent light and 12 h darkness, and spore suspensions for inoculation were prepared by flooding the cultures with 15 ml sterile distilled water and dislodging the conidia with a disposable hockey stick cell spreader. Spores were quantified with a Fuchs Rosenthal hemocytometer.

Pea seeds were surface sterilized in 70% ethanol for 5 min and rinsed with distilled water before planting four seeds into 500 ml pots filled with previously autoclaved sand. Following sowing the pots were inoculated with 2 x 10^4^ spores g^-1^ substrate. Controls were left non-inoculated. The experiment was arranged in a completely randomized design with three replicates. The pots were kept in the greenhouse at 19/16°C day/night temperature and 16 h photoperiod (provided with 400 W high-pressure sodium lamps). Pots were watered daily with tap water and additionally fertilized with the complex N:P:K fertilizer Wuxal Super (8:8:6 + microelements). A total of 100 mg of N l^-1^ of substrate was divided in two equal portions and added 10 and 15 days after sowing.

After 21 days, plants were removed from pots, and the roots were separated from the above ground biomass. Above ground plant parts of each pot were weighed and dried at 105°C until constant weight was achieved. Roots were washed under running tap water, and root rot severity was assessed using a visual 0–8 score scale based on external and internal root tissue discoloration levels adopted from Pflughöft et al. [46]. The external disease severity was rated as follows: disease severity rating (DSR) 0 = no symptoms, 1 = streaks at the transition zone of epicotyl to hypocotyl, 2 = brown lesions cover up to 50% of root perimeter, 3 = brown-black lesions cover 51 to 99% of root perimeter, 4 = black lesions cover 100% of stem perimeter, 5 = black lesions spread up to 30–49% of the tap root, 6 = black lesions spread up to 50 to 70% of the tap root, 7 = black lesions spread > 70% of the tap root, 8 = dead plant. The roots were then cut transversally across the lesions and internal disease severity was rated, where 0 = no visible symptoms, 1 = epidermis/rhizodermis is brown to black, 2 = brown discoloration of cortical tissues, 3 = cortical tissues is partially black, but the center and endodermis are still healthy, 4 = cortex tissue is completely black, 5 = cortex tissue begins to rot (bursting of epicotyl or rhizodermis on the root), 6 = cortex tissue is completely rotten, 7 = shedding of the cortex tissue and endodermis, and 8 = dead plant. A disease severity index (DI) between 0 and 100 was calculated for each pot using the following formula:

$$DI=\frac{Ʃ(SR\;x\;NR)}{Nt\;x\;8}*100$$ (1)

Here, SR denotes the mean external and internal disease severity rating (DSR), NR denotes the number of infected plants having that DSR and Nt is the total number of plants assessed.

**Fig 1:**
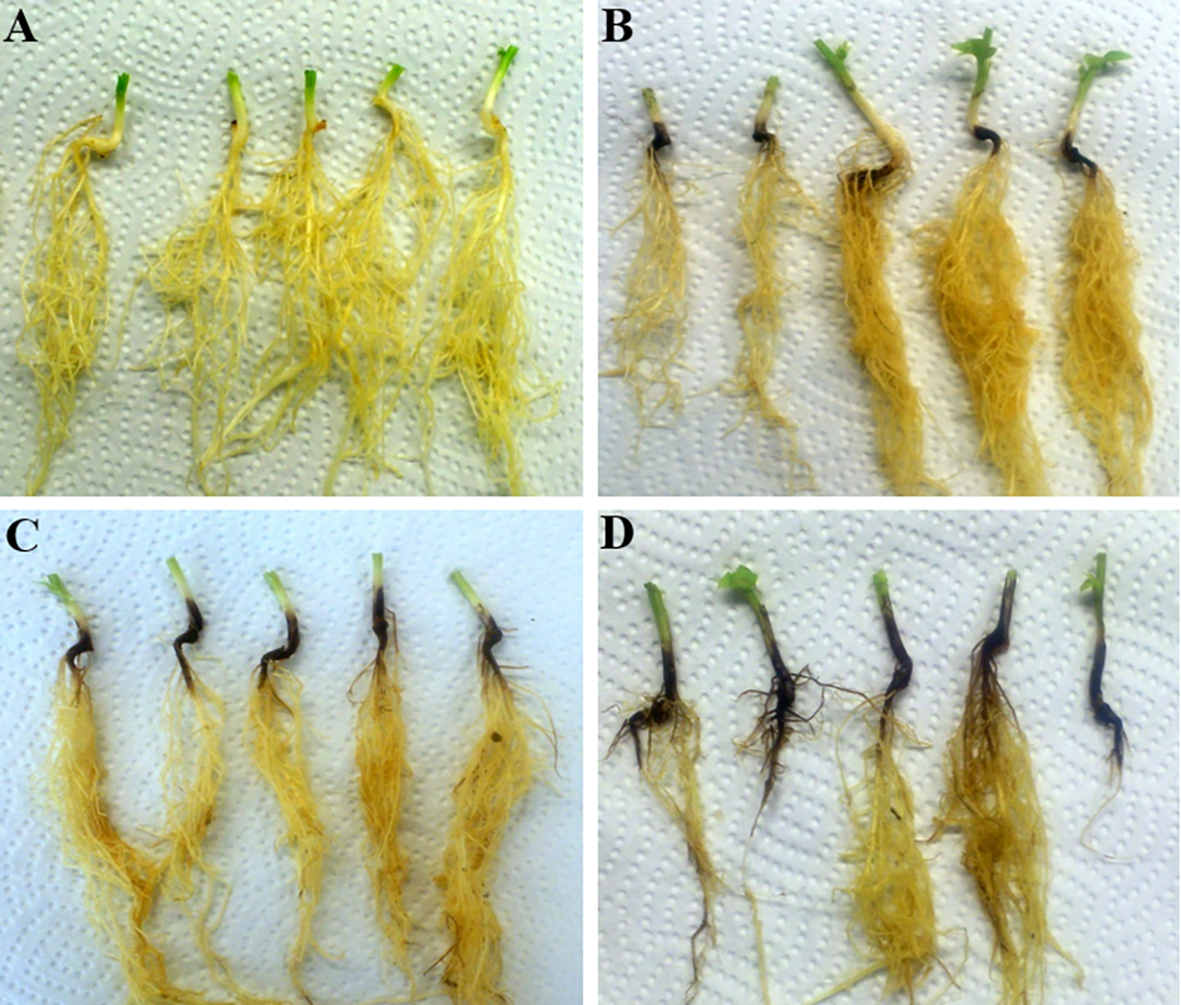
Classification of isolates into four distinct aggressiveness classes based on the root rot severity symptoms. (A) non-aggressive (DI = 0–15), (B) weakly aggressive (DI = 16–30), (C) moderately aggressive (DI = 31–70) and (D) highly aggressive (DI = 71–100).

Four distinct aggressiveness classes were assigned based on the gradual increase of severity of symptoms following inoculation. When the DI of inoculated plants was in the same range as the DI of the un-inoculated control (DI = 0–15), the isolate was classified as non-aggressive (approximately correspond to DSR of 0 to 1); DI = 16–30 –weakly aggressive (DSR ≈ 2 to 3); DI = 31–70 –moderately aggressive (DSR ≈ 4 to 6); and DI = 71–100 –highly aggressive (DSR ≈ 7 to 8) (Fig 1).

Within each species of *Fusarium* tested, up to ten different treatments were selected at random, and fungi were re-isolated from the roots using the protocol described above in order to confirm that infection was the result of the inoculated species.

### Data analysis

For each *Fusarium* spp., isolation frequencies were calculated for each respective host, year and site as follows:

$$Frequency\;(F)=\frac{n}{N}*100$$ (2)

Here, n denotes the number of root samples infected with a species and N denotes the total number of root samples.

In addition, *Fusarium* spp. relative densities (RD) averaged over the two years were estimated at the site level as the number of isolates of each species (x 100) divided by the total number of *Fusarium* isolates.

As most data were not normally distributed and some groups had unequal variances, results were evaluated with nonparametric analyses. Year, host, and site effects on mean disease severity ratings and on isolation frequencies of *Fusarium* species were tested using the non-parametric ranking procedure of the Kruskal-Wallis test in R statistical software (version 3.3.0, R Core Team, [47] using the package agricolae [48]. If significant treatment effects were observed (*P* < 0.05), mean ranking values were separated with the Kruskal multiple comparison test [49]. As there was no significant difference in *Fusarium* community composition (ANOSIM analysis, see text below), data on isolation frequencies of *Fusarium* spp. isolation from the samples collected at the southern and central German sites were pooled and analyzed together. Species that occurred less than ten times in the survey were excluded from statistical analyses. The Benjamini and Hochberg [50] stepwise adjustment of *P* values was used to control false discovery rate (FDR) and reduce type I errors in a post hoc procedure.

To test for differences in *Fusarium* community composition, one way ANalysis Of SIMilarities (ANOSIM, [51] was performed on pooled abundance data from both years, using the PRIMER v7 software for windows (Plymouth Routines in Multivariate Ecological Research, Plymouth Marine Laboratory, UK). Bray—Curtis dissimilarity matrices of the fourth square root transformed data were constructed and applied to compare ranked similarities for differences within and between previously defined groups using 10.000 random permutations.

For the greenhouse experiment, overall mean effects of *Fusarium* spp. and effects of single isolates on the pea biomass were expressed as percentage of change in fresh weight per plant (FW change) relative to non-inoculated control, using the following formula:

$$FW\;change\;(\%)=\frac{X_{2}-X_{1}}{X_{1}}*100$$ (3)

Here, X_2_ denotes mean fresh weight per plant in g of inoculated treatment and X_1_ denotes mean fresh weight per plant in g of the non-inoculated control.

Differences in root rot severity (disease index values) and effects on fresh weight of *Fusarium* spp. averaged over isolates were analyzed using the Kruskal-Wallis test followed by the Kruskal multiple comparison test as described above. Differences among single isolates were tested separately for each *Fusarium* spp. by comparing mean rank sums from inoculated treatments with non-inoculated controls and the mean effect of the two pathogenic *F*. *solani* f. sp. *pisi* isolates (positive control) using Dunn's multiple comparison test with one control [52]. Effect of isolate was considered significant with an FDR adjusted *P* value < 0.05. Simple linear regression analysis was performed for each *Fusarium* species to estimate yield losses associated with root rot severity [53].

## Results

### Root disease assessment of field plants

Mean root rot severity ratings of the sampled plants were very low and similar within hosts across all sites in both years. The lowest symptom severities were observed on both clover species with mean disease severity ratings of 0.7 and 0.5 for subterranean and white clover, respectively. Vetches, on average, had slightly higher symptom severity with a mean disease rating of 1.7 (Table 2).

**Table 2:** Mean root rot severity ratings^1^ for each plant species at sampled fields in 2013 and 2014.

	Switzerland		Central Germany		Southern Germany	Sweden		Overall mean
	2013	2014	2013	2014	2013	2013	2014	
Subterranean clover	0.4 b^2^	1.1 a	0.4 b	0.2 c	0.3 bc	-	-	0.7 B^3^
White clover	-	-	0.0 b	0.2 c	-	1.0 a	1.1 a	0.5 C
Vetch	-	1.8 a	1.9 a	-	0.8 c	-	1.5 b	1.7 A

^1^Root rot disease severity was evaluated using a 0–5 rating scale. Total number of plants assessed per site and year are given in Table 1.

^2^Means within row followed by different lower-case letters indicate significant differences in disease severity ratings among the sites and sampling years within the same plant species.

^3^Means followed by different upper-case letters indicate significant differences in overall mean severity ratings among the plant species averaged over the sites and the two sampling years (Kruskal post hoc test, *P* < 0.05).

### Host and effect of sampling time on *Fusarium* species

Out of 1480 roots analyzed a total of 670 *Fusarium* isolates were obtained over the two sampling periods. Of these, 388 isolates were obtained in 2013 with 273 isolated from subterranean clover (n = 400), 3 from summer vetch (n = 80), 29 from winter vetch (n = 80) and 83 from white clover (n = 200). In 2014, a total of 282 isolates were obtained with 147 from subterranean clover (n = 320), 124 from winter vetch (n = 280) and 11 from white clover (n = 120). The recovered isolates represented the following 14 species (in the order of frequency of occurrence): *F*. *oxysporum*, *F*. *avenaceum*, *F*. *solani*, *F*. *equiseti*, *F*. *acuminatum*, *F*. *redolens*, *F*. *culmorum*, *F*. *graminearum*, *F*. *tricinctum*, *F*. *crookwellense*, *F*. *poae*, *F*. *sambucinum*, *F*. *sporotrichioides* and *F*. *torulosum* (S2 Table).

**Fig 2:**
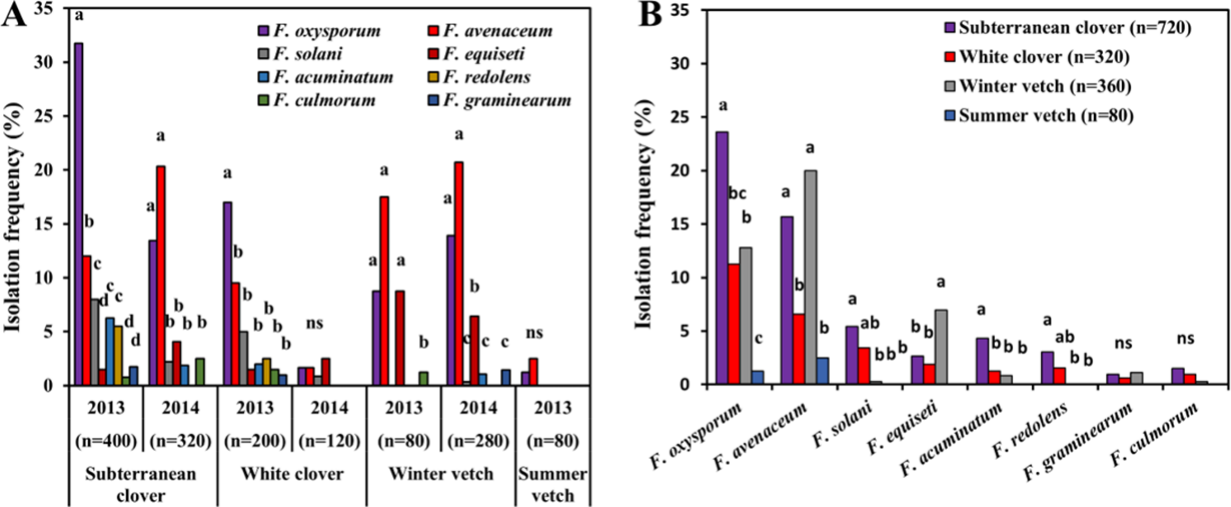
Isolation frequencies of the most common *Fusarium* spp. recovered from the roots of four hosts. (A) Effects of the sampling year and (B) the host plant averaged over the two sampling years. Means followed by different letters indicate significant differences in isolation rates within host plant and respective year (A), and among different hosts for respective species of *Fusarium* (B) (Kruskal post hoc test, *P* < 0.05). n = number of roots assessed.

*Fusarium oxysporum* and *F*. *avenaceum* were the most frequently isolated species in both, 2013 and 2014 (Fig 2A). Isolation frequencies of *F*. *oxysporum* were significantly greater on subterranean and white clover in 2013 (*P* < 0.05) compared to all other species, whereas in 2014, *F*. *avenaceum* was most comonly recovered from the roots of subeterranean clover and both vetches. *Fusarium oxysporum* was the only species occurring regularly at all sites and on all plant hosts in both years, while *F*. *avenaceum* was not detected in the roots of white clover from Sweden, or in subterranean clover from Italy in 2014. Particularly low percentages of *Fusarium* isolates were recovered from white clover roots in 2014 and the roots of summer vetch in 2013 (Fig 2A, S2 Table).

Averaged over the two sampling years, mean isolation frequency of *Fusarium* spp. was significantly higher in 2013 (51.1%) than in 2014 (39.2%) (*P* = 0.004). This was mostly due to a decrease in frequencies of *F*. *oxysporum* (22.2%, 2013, and 11.7%, 2014, *P* < 0.001), *F*. *solani* (5.5%, 2013, 1.3%, 2014, *P* = 0.001), *F*. *acuminatum* (3.8%, 2013, 1.3%, 2014, *P* = 0.002) and *F*. *redolens* (3.6%, 2013, 0%, 2014, *P* < 0.001) between the two years. In contrast, isolation rates for *F*. *avenaceum* (10.9%, 2013, 17.4%, 2014, *P* = 0.072) and *F*. *equseti* (2.1%, 2013, 4.7%, 2014, *P* = 0.20) increased between the two years, although these changes were not statistically significant.

The main trends observed for combined data averaged over the years were similar to those observed for data plotted per year (Fig 2B). For example, *F*. *oxysporum* was significantly more frequent on subterranean clover than on all other hosts, while frequency of *F*. *avenaceum* was significantly higher on subterranean clover and winter vetch compared with white clover and summer vetch. Frequency of isolation of *F*. *equiseti* was greater on winter vetch compared to all other hosts, whereas *F*. *solani* and *F*. *acuminatum* were more common on the roots of subterranean clover (Fig 2B). The remaining *Fusarium* species identified occurred only sporadically without apparent preference for any host.

### Site variations in populations of *Fusarium* spp.

Out of the 670 *Fusarium* isolates, 336 (50.1%) originated from Switzerland (n = 360 roots), 180 (26.9%) from Germany (n = 640 roots), 117 (17.5%) from Italy (n = 360 roots), and 37 (5.5%) from Sweden (n = 120 roots). The one-way ANOSIM test showed that the *Fusarium* communities at the Swiss site were significantly different from those of the German, Italian, and Swedish sites (*P* < 0.001), with the latter three not significantly different from each other. However, the mid-range value of R (= 0.52) for the Swiss-Swedish comparison contrasted with much lower R values for the Swiss-German and Swiss-Italian comparisons (R = 0.10 and 0.26, respectively), implies that the separation was moderately strong for the former and rather weak for the latter two comparisons. Lack of strong separation suggests that samples within the sites were just as similar in *Fusarium* community composition as samples between the sites (Table 3). No significant differences were found between sites according to two-way nested ANOSIM (plant species within sampling sites).

**Table 3:** Differences among sites in the *Fusarium* community composition. R and P-values obtained for every pair of sampling site using one-way ANOSIM performed on pooled abundance data from both years.

	Switzerland		Germany		Sweden	
	R value	*P* value	R value	*P* value	R value	*P* value
**Germany**	**0.1090**	**0.0001**				
**Sweden**	**0.5210**	**0.0001**	-0.0180	0.6660		
**Italy**	**0.2640**	**0.0001**	0.0080	0.2000	0.0140	0.3130

The most prevalent species isolated were *F*. *oxysporum* and *F*. *avenaceum* accounting for 68.8% of all fusaria (37.8% *F*. *oxysporum*; 31.0% *F*. *avenaceum*; a total of 461/670 isolates). These two species were identified as the most prevalent based on the mean frequencies of isolation averaged over the years and the relative densities of occurrence *i*.*e*. percentage of the total number of *Fusarium* isolates obtained at each site (Table 4). However, the main trends observed for these two species differed. *Fusarium oxysporum* dominated the populations in Italy and Sweden with relative densities (RD) of 61.5% and 51.4%, respectively, while RDs of *F*. *avenaceum* were only 17.1% and 8.1% at these sites, respectively. In contrast, in Switzerland and Germany the RDs of the two pathogens were similar (Table 4). Other species such as *F*. *solani*, *F*. *equiseti* and *F*. *acuminatum* were less common and represented 1 to 14% of the isolates. The former two species were more frequent at the German, Swedish and Swiss sites, whereas *F*. *acuminatum* was more common in Italy (*P* < 0.05). Also present, but less frequent, were *F*. *redolens* and *F*. *graminearum* mainly recovered from the Swiss site (*P* < 0.05). The species *F*. *culmorum* was not isolated from Italy. Most remaining species found were represented by a few isolates only (Table 4).

**Table 4:** Frequencies (%) of isolation (F) and relative densities (RD, %) of *Fusarium* species for each studied site averaged over the hosts and the two sampling years.

*Fusarium* species	Italy			Switzerland			Germany			Sweden		
	F	RD	n^1^	F	RD	n	F	RD	n	F	RD	n
*F*. *oxysporum*	20.0 a^2^ B^3^	61.5	72	31.4 a A	33.6	113	7.7 ab C	27.2	49	15.8 a BC	51.4	19
*F*. *avenaceum*	5.6 b B	17.1	20	36.1 a A	38.7	130	8.6 a B	30.6	55	2.5 b B	8.1	3
*F*. *solani*	0.6 c B	1.7	2	6.1 b A	6.5	22	3.6 bc A	12.8	23	3.3 b AB	10.8	4
*F*. *equiseti*	0.8 c B	2.6	3	6.7 b A	7.1	24	3.0 cd B	10.6	19	3.3 b AB	10.8	4
*F*. *acuminatum*	4.7 b A	14.5	17	3.1 bc AB	3.3	11	1.4 d B	5.0	9	0.8 b B	2.7	1
*F*. *redolens*	0.8 c B	2.6	3	5.0 bc A	5.4	18	0.3 d B	1.1	2	3.3 b AB	10.8	4
*F*. *graminearum*	-	-	-	2.8 bc A	3.0	10	0.5 d B	1.7	3	-	-	-
*F*. *culmorum*	-	-	-	1.1 c AB	1.2	4	1.6 cd A	5.6	10	0.8 b B	2.7	1
*F*. *tricinctum*	-	-	-	0.6 c NS	0.6	2	0.5 d	1.7	3	-	-	-
*F*. *poae*	-	-	-	0.3 c NS	0.3	1	0.2 d	0.6	1	-	-	-
*F*. *sporotrichioides*	-	-	-	0.3 c NS	0.3	1	-	-	-	-	-	-
*F*. *sambucinum*	-	-	-	-	-	-	0.6 d	2.2	4	-	-	-
*F*. *crookwellense*	-	-	-	-	-	-	0.3 d	1.1	2	-	-	-
*F*. *torulosum*	-	-	-	-	-	-	-	-	-	0.8 b B	2.7	1

^1^n = number of recovered isolates.

^2^Means in columns followed by different lower-case letters indicate significant differences in mean *Fusarium* spp. isolation frequencies (F) within the respective site.

^3^Means in rows followed by different upper-case letters indicate significant differences between the sites in mean isolation frequencies (F) within the same *Fusarium* species (Kruskal post hoc test, *P* < 0.05). NS = non-significant.

### Aggressiveness of *Fusarium* species on pea

*Fusarium avenaceum* caused the most severe root rot symptoms on pea (mean Disease Index, DI = 82), followed by *F*. *oxysporum* (DI = 39), *F*. *solani* (DI = 34) and *F*. *tricinctum* (DI = 25). *Fusarium equiseti* and *F*. *acuminatum* caused little root damage and overall were found to be non- or only weakly aggressive, with mean DIs of 13 and 17, respectively (Table 5). Overall, only *F*. *avenaceum* and *F*. *oxysporum* caused significant reductions in pea plant biomass comapred to the non-inoculated control (mean reduction = 74% and 19%, respectively). Also, a highly significant linear relationship was found between root rot severity and biomass reduction especially for *F*. *avenaceum* (R^2^ = 0.91, *P* < 0.001). Statistically significant, but weak relationships between the two parameters were also observed for *F*. *oxysporum* and *F*. *solani*, while mean root rot severity for *F*. *equiseti*, *F*. *tricinctum* and *F*. *acuminatum* was low and could not be correlated with biomass reduction on the three-week old pea plants. Compared to the *F*. *solani* f. sp. *pisi* positive control, significantly higher mean root rot severity and/or reduction of plant biomass was observed only for *F*. *avenaceum* and *F*. *oxysporum* (Table 5).

**Table 5:** Mean root rot severity (DI), correlation between DI and fresh weights, and changes in fresh weights of three week old pea plants caused by six *Fusarium* species.

Pathogen (n)^1^	Mean DI^2^	Mean FW change (%)^3^	Correlation coefficient^4^	FW change (%)	
				Maximum	Minimum
*F*. *oxysporum* (16)	39.0 c^5^	-19.3 b	0.165**^6^	-33.4	-10.2
*F*. *avenaceum* (16)	82.4 a	-74.0 a	0.910***	-100.0	-13.8
*F*. *solani* (24)	32.6 d	-3.5 c	0.080*	-39.2	+10.1
*F*. *equiseti* (4)	12.8 f	+5.0 d	0.003	-4.7	+14.1
*F*. *acuminatum* (8)	16.5 f	+7.6 d	0.056	-6.3	+22.0
*F*. *tricinctum* (2)	25.3 e	+1.0 cd	0.003	-2.5	+4.4
*F*. *solani* f. sp. *pisi* (2)	48.9 b	0.0 cd	-	-1.1	+1.0
Control	6.3 e	0.0 cd	-	-	-

^1^n = total number of isolates tested.

^2^Disease severity is expressed as Disease Index (DI), where DI = 0–15 –non-aggressive; DI = 16–30—weak aggressiveness; DI = 31–70—moderate aggressiveness; DI = 71–100—high aggressiveness.

^3^Changes in fresh weights are expressed as percentage change in fresh weight of inoculated treatments relative to non-inoculated control.

^4^Correlation between DI and fresh weight change.

^5^Different letters indicate significant differences according to Kruskal multiple comparison test (*P* < 0.05).

^6^***, **, *: *P* < 0.001, 0.01 and 0.05, respectively. Data presented are means across isolates for each *Fusarium* spp. tested.

**Fig 3:**
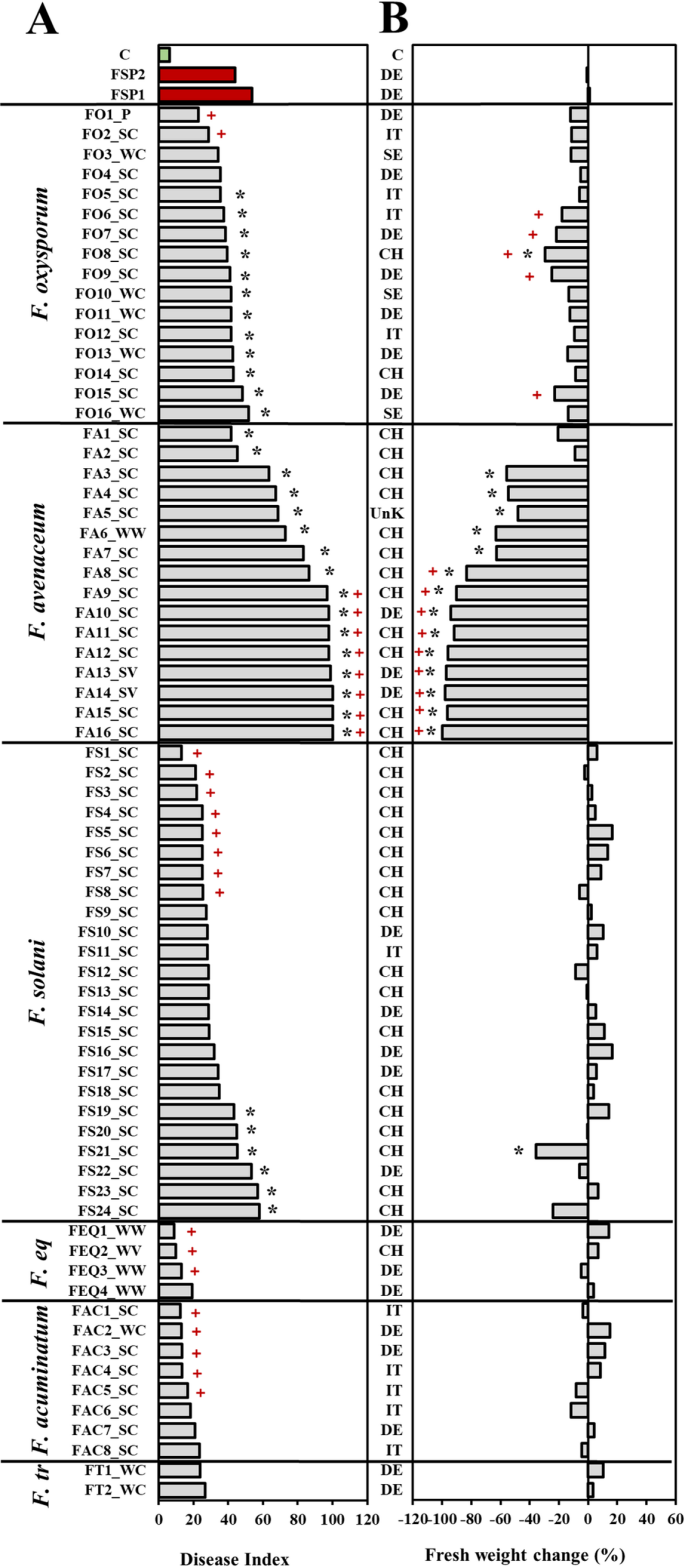
**Effects of *Fusarium* spp. isolates on pea root rot severity (A), and fresh weight (B).** Effects on the fresh weights are expressed as percentage change relative to the non-inoculated control. Disease severity is expressed as Disease Index (DI), where DI = 0–15 –non-aggressive; DI = 16–30—weak aggressiveness; DI = 31–70—moderate aggressiveness; DI = 71–100—high aggressiveness. F. eq *= F*. *equiseti*, F. tr = *F*. *tricinctum*. The letters in the suffix of each isolate ID number represent the host plant from which isolates were collected, where SC = subterranean clover, WC = white clover, WV = winter vetch, SV = summer vetch, WW = winter wheat. Geographical origin of the isolates are denoted by the country of origin, where IT = Italy, chittCH = Switzerland, DE = Germany, SE = Sweden, and UnK = unknown. C = non-inoculated control. Asterisks next to the bars indicate significant difference from the non-inoculated control plants. The + symbols indicate significantly different from the mean effect of two *F*. *solani* f. sp. *pisi* isolates (FSP1 and FSP2) used as positive control. Dunn's multiple comparison test with one control (*P* < 0.05). Data presented are means of three replicate pots.

Among individual isolates within *Fusarium* species, significant variation in aggressiveness occurred for both, root rot severity and reductions of biomass (*P* < 0.05). Root rot severity following inoculation with the majority of *F*. *oxysporum* isolates differed significantly from the non-inoculated control (Fig 3A). Among the 16 isolates tested, two (2/16, 13%) were classified as weakly and 14 (87%) as moderately aggressive. While all *F*. *oxysporum* isolates caused biomass reductions, in comparison to the non-inoculated control the difference was statistically significant only for isolate FO8. This isolate was recovered from subterranean clover roots, and caused a biomass reduction of 34% (Fig 3B). The lowest DI was observed for the isolate FO1 from pea, but biomass was still reduced by 17%. Compared to the positive control, none of the *F*. *oxysporum* isolates differed significantly in root rot severity, however, five of the isolates (31%) were still classified as more aggressive than *F*. *solani* f. sp. *pisi* due to the significant reduction of plant biomass (Fig 3A and 3B).

All of the *F*. *avenaceum* isolates tested differed significantly in root rot severity from the non-inoculated control (Fig 3A). Disease severity indices ranged from 41 to 100, and biomass reductions ranged between 20 and 100% compared to the non-inoculated control. Among the 16 isolates tested, 2 isolates (13%) recovered from subterranean clover roots were classified as moderatelly aggressive, whereas the remaining 14 isolates (87%) were classified as highly aggressive. The isolates FA3, FA4 and FA5 induced moderately severe symptoms, but were classified as highly aggressive due to the high biomass reductions of more than 50% relative to the non-inoculated control plants (*P* < 0.05). Nine isolates (56%) of this species were more aggressive than the *F*. *solani* f. sp. *pisi* positive control (Fig 3A and 3B).

The effects of the *F*. *solani* isolates were highly variable. Among the 24 isolates tested, one isolate (4%) was classified as non-aggressive, 14 isolates (58%) were weakly aggressive and 9 isolates (38%) were moderately aggressive. However, despite low to moderate root disease severities observed, 11 out of 24 isolates (46%) tested increased biomass of pea by up to 10% compared to the non-inoculated control. The most aggressive isolate of *F*. *solani* was recovered from subterranean clover roots (FS21), and caused moderate disease severity with a biomass reduction of 39% compared to the non-inoculated control (*P* < 0.05). Eight isolates of this species were found to be less aggressive than the *F*. *solani* f. sp. *pisi* positive control, whereas the remaining isolates were similarly aggressive.

The isolates of *F*. *equiseti* and *F*. *acuminatum* were classified as non- to weakly aggressive, often causing increases in biomass, ranging from 9 to 20% compared to the non-inoculated control. Finally, the isolates of *F*. *tricinctum* were weakly aggressive and had no significant effect on root rot severity or pea biomass, compared to both, the negative and positive control treatments (Fig 3A and 3B).

## Discussion

We identified 14 *Fusarium* spp. from asymptomatic roots of subterranean and white clover and from winter and summer vetch roots with slight discolorations. The root discolorations observed in vetch were due to mycorrhiza infections based on microscopical results. The fact that all of the isolates collected were recovered from mostly asymptomatic clover and vetch roots in the field suggests that they were endophytes with respect to their original hosts [54]. *Fusarium oxysporum* and *F*. *avenaceum* were ubiquitous, accounting for 68.8% of total isolation percentage and occurring in most of the sites and on all four hosts. Less frequently found species were *F*. *solani*, *F*. *equiseti*, *F*. *acuminatum*, *F*. *redolens*, *F*. *culmorum*, *F*. *graminearum*, *F*. *tricinctum*, *F*. *crookwellense*, *F*. *poae*, *F*. *sambucinum*, *F*. *sporotrichioides* and *F*. *torulosum*. The *Fusarium* communities found at each site were dominated by similar species. Although the differences in community structure of the Swiss site from all other sites were statistically signifcant only the separation of the Swiss and Swedish sites was supported by moderately high R values. Thus, overall, environment and host plant effects on the *Fusarium* community composition is considered low with the main effects being on the isolation frequencies of fungal species found.

When tested on pea, 33 out of the 65 isolates of six *Fusarium* species recovered from the clover and vetch species caused significantly higher disease severity symptoms than the negative controls. The same was true for the one *F*. *avenaceum* isolate originating from wheat. Most of these isolates were at least as aggressive as *F*. *solani* f. sp. *pisi* that served as positive controls. Nine of the *F*. *avenaceum* and five of the *F*. *oxysporum* isolates were found to be more aggressive than the positive controls, not only in terms of disease symptom development, but also for their effects on pea biomass. On the one hand, this demonstrates the ability of the most abundant species/isolates to switch between an endophytic and pathogenic lifestyle depending on the host. On the other hand, the data also confirms the role of *F*. *avenaceum* as a severe root rot pathogen of pea [55,25].

*Fusarium avenaceum* is a wide host range pathogen that dominates the *Fusarium* Head Blight (FHB) complex of small grain cereals in Northern Europe [56]. The fungus also causes economically important diseases on a range of different crops, such as soybean, maize, barley and oat [57–59]. *Fusarium avenaceum* produces a range of potent mycotoxins such as moniliformin, enniatin, and beauvericin [60], which can cause severe feed refusal disorders in sheep [61–63]. The emerging importance and high pathogenic potential of *F*. *avenaceum* on pea and other legumes points to the need to examine the role of these secondary metabolites in the long and short term strategies of the fungus in colonizing legumes as well as in occupying diverse agro-ecological niches.

The emergence of *F*. *avenaceum* as an important pathogen, particularly of field pea, is relatively recent. This has mainly been attributed to climate change and lack of host specificity of the pathogen that enables persistence of the fungus even under diverse crop rotations [55,64]. Although, the precise combination of climatic and soil conditions favoring this pathogen remain unknown, in our study *F*. *avenaceum* was prevalent at the Swiss, German and Italian sites, whereas in Sweden the fungus occurred only rarely. This rare occurrence of *F*. *avenaceum* at the Swedish site may be due to the significantly higher soil organic matter (SOM) content of the site compared to all others. For example, Yli-Mattila et al. [65] and Lager and Gerhardson [66] identified *F*. *avenaceum* as the dominating pathogenic fungus on red clover in Finland and on red clover and white clover in central Swedish organic fields, respectively. However, the authors did not provide sufficient soil and climatic data that would allow to compare their study with ours. Similarly, according to Wong et al. [67] and Barbetti et al. [68], *F*. *avenaceum* is one of the most common species isolated from the roots of subterranean clover dominated pastures in western Australia. Low SOM and nutrient-poor soils across many parts of these Australian regions were found to be favourable to *F*. *avenaceum* in particular, severely limiting the time that subterranean clover can be used in rotations. In our study from the total of 720 subterranean clover plants sampled in the two years, 420 yielded *Fusarium* species with the maximum isolation rates found for *F*. *avenaceum* in Switzerland (36%). However, there were no obvious disease symptoms due to *F*. *avenaceum*. This suggests that endophytic behaviour in the field may depend on pedoclimatic conditions and potentially on inoculum pressure. More intensive sampling over the course of several years would be necessary to determine the temporal changes in the pathogen populations and how these relate to soil specific conditions and changes in crops over the course of rotations.

The prevalence of *F*. *oxysporum* across the broad range of ago-climatic regions sampled confirms its wide adaptation and is consistent with work of Lager and Gerhardson [66], Nan [69] and Rufelt [70], who reported that *F*. *oxysporum* was the predominantly isolated *Fusarium* sp. from diseased roots of red clover in Sweden and New Zealand. *Fusarium oxysporum* together with *F*. *solani* are commonly isolated from symptomatic pea and soybean (*Glycine max*) roots in Germany, Sweden, Canada and the USA [23,25,46,71,72]. However, aggressiveness among isolates of both species is highly variable. For example, *F*. *oxysporum* isolates from diseased pea roots in North Dakota were only weak pathogens on pea [25], while in Scandinavia some highly aggressive isolates were found, which induced severe root rot and wilt symptoms in greenhouse bioassays [73,23]. Arias et al. [74] reported similar results for the growth and yield of soybeans after root inoculation with *F*. *oxysporum* and *F*. *solani* isolates. Our results are in agreement with these studies and reveal similar variation in aggressiveness among the *F*. *oxysporum* and in particular the *F*. *solani* isolates tested. As suggested by Chittem et al. [25], isolates of any of the two species alone may not be effective at causing root rot severity, rather they are a part of the root rot complex contributing to overall disease severity. As *F*. *oxysporum* and *F*. *solani* represent phylogenetically diverse species complexes comprising of saprophytic, endophytic and pathogenic strains [33], the high pathogenic variability of the tested isolates on pea observed in our study is not a surprising.

The isolates of *F*. *tricinctum*, *F*. *equiseti* and *F*. *acuminatum* caused some root discoloration on pea, but no reductions in fresh weights, some even increased biomass considerably. *Fusarium tricinctum* is generally considered a soil saprophyte and an opportunistic pathogen in temperate regions mainly associated with wheat, where the fungu*s* is part of the complex causing Fusarium Head Blight (FHB) of small grain cereals in Europe and North America [56]. The species has not been reported as part of the root rot complex of pea, however, recent studies suggest that *F*. *tricinctum* could be an important pathogen of soybean [75]. *Fusarium equiseti* and *F*. *acuminatum* are naturally occurring endophytes and opportunistic pathogens in diverse ecosystems and are able to colonize the roots of various hosts [33]. A recent study of Šišić et al. [76] on the potential ecological role of *F*. *equiseti* revealed that three of the four *F*. *equiseti* isolates tested in our study here have the potential to act as biocontrol agents inhibiting *F*. *avenaceum* and *Peyronellaea pinodella* development in the root system of pea plants and suppress associated disease.

In conclusion, our results demonstrate the ability of many *Fusarium* species that are pathogenic to pea to asymptomatically reside in clover and vetch plants that may thus serve as a reservoir of inoculum. For *F*. *avenaceum* this has also been shown to be true for various weeds [35]. We also provide new information about the diversity, relative frequencies and aggressiveness of *Fusarium* spp. associated with white and subterranean clover and vetch that are popular cover crops and living mulch species in Europe. Thus, some of the major pathogens of various leguminous and non-leguminous crops are characterized by high ecological plasticity. They have the ability to endophytically colonize the hosts studied and the potential to cause yield losses in subsequent main legume grain crops such as pea.

## Supporting information

S1 Table Root rot disease assessment of field plants. (XLSX)

S2 Table Number of *Fusarium* isolates recovered. (XLSX)

S3 Table Aggressiveness of *Fusarium* spp. in greenhouse. (XLSX)
